# Modeling of SPM-GRU ping-pong ball trajectory prediction incorporating YOLOv4-Tiny algorithm

**DOI:** 10.1371/journal.pone.0306483

**Published:** 2024-09-06

**Authors:** Fuxing He, Yongan Li

**Affiliations:** 1 School of Physical Education, Qiongtai Normal University, Haikou, China; 2 School of Physical Education, Hainan Normal University, Haikou, China; Zhejiang Normal University, CHINA

## Abstract

The research aims to lift the accuracy of table tennis trajectory prediction through advanced computer vision and deep learning techniques to achieve real-time and accurate table tennis ball position and motion trajectory tracking. The study concentrates on the innovative application of a micro-miniature fourth-generation real-time target detection algorithm with a gated loop unit to table tennis ball motion analysis by combining physical models and deep learning methods. The results show that in the comparison experiments, the improved micro-miniature fourth-generation real-time target detection algorithm outperforms the traditional target detection algorithm, with the loss value decreasing to 1.54. Its average accuracy in multi-target recognition is dramatically increased to 86.74%, which is 22.36% higher than the original model, and the ping-pong ball recognition experiments show that it has an excellent accuracy in various lighting conditions, especially in low light, with an average accuracy of 89.12%. Meanwhile, the improved model achieves a processing efficiency of 85 frames/s. In addition, compared with the traditional trajectory prediction model, the constructed model performs the best in table tennis ball trajectory prediction, with errors of 4.5 mm, 25.3 mm, and 35.58 mm. The results show that the research trajectory prediction model achieves significant results in accurately tracking table tennis ball positions and trajectories. It not only has practical application value for table tennis training and competition strategies, but also provides a useful reference for the similar techniques application in other sports.

## Introduction

In recent years, as a sport widely participated in by the world, the improvement of training methods of table tennis has gradually received attention, especially in high-level competitions, the demand for dynamic trajectory and behavior analysis of the ball is growing. However, the existing table tennis trajectory prediction technology mostly relies on traditional video analysis methods, although these methods can provide certain trajectory information, it is often difficult to meet the needs of high-level training and competition in real-time and accuracy. In addition, these systems often require expensive hardware support and complex setup, limiting their general application [1]. Advanced computer vision and deep learning technologies are reshaping training methods and competition strategies in predicting and analyzing table tennis trajectories. Especially in the dynamic trajectory prediction (TP) of table tennis, traditional methods are gradually unable to meet the needs of high-level training and competition, and applying deep learning to table tennis motion analysis has become a hot research topic [2]. In this context, the deep learning method that integrates the physical model (SPM) of table tennis provides a new approach for achieving accurate prediction of table tennis trajectories. SPM not only describes the kinematic characteristics of table tennis, such as bounce, rotation, and flight path, but also includes aerodynamic factors, making it an important foundation for TP. As an efficient object detection algorithm, You Only Look Once (version 4 Tiny, YOLOv4 Tiny) is widely used in multiple real-time video processing fields due to its lightweight network structure and high real-time performance [3]. Although its performance is slightly lower than the more complex YOLO version, YOLOv4 Tiny significantly reduces the demand for computing resources while maintaining high accuracy, making it an ideal choice for real-time sports analysis; Gated Recurrent Unit (GRU) is an efficient recurrent neural network (RNN) that excels in processing time series data [4, 5]. To solve these problems, a ping pong trajectory prediction model based on improved YOLOv4-Tiny algorithm and GRU is proposed. The model aims to improve the capture and prediction accuracy of dynamic trajectories of ping-pong balls through deep learning technology, while keeping the system low cost and high practicality. By processing video data in real time, the model is able to accurately identify and predict the position and movement trajectory of the ping-pong ball, thus providing immediate and accurate feedback to coaches and players to help them optimize training and competition strategies. The research content has four parts. The Part 1 analyzes the relevant research on YOLOv4 Tiny algorithm and table tennis training system; In the second part, the study first introduces the improvement process of YOLOv4 Tiny algorithm, and then constructs a table tennis TP model based on SPM-GRU. The Part 3 verifies the effectiveness and reliability of the research model through experimental design and data analysis. Part 4 summarizes the paper and make an outlook of the future.

## Related works

In the study of YOLOv4, Saputra APK [6] addresses the increasing problem of waste categorization with deep learning algorithms for object detection using YOLOv4 and modified YOLOv4-Tiny models. The two models are evaluated under different input conditions and webcams, and the results show that YOLOv4-Tiny, on the other hand, is more efficient in terms of computational speed. Xiang et al. [7] designed an intelligent engineering vehicle detection built on the YOLOv4-Tiny dedicated to raw material warehouse scenarios. The method performs well in terms of both accuracy and speed and is suitable for intelligent monitoring and management of engineering vehicles. Gao et al. [8] developed a feature-enhanced YOLOv4 Tiny-based detection method for the recognition challenges of electrical components in smart grids. Experiments show that the proposed YOLOv4 tiny significantly improves the detection accuracy while maintaining the real-time processing capability. Li et al. [9] developed a real-time vehicle detection and tracking system based on the YOLOv4 algorithm. The system achieves efficient detection, recognition and tracking functions for vehicles in surveillance cameras. Experiments showed that the system achieved 96.30% accuracy and 94.17% overall accuracy in vehicle tracking. In this paper, Zhu and Zhao [10], we concentrate on the mask target detection technique based on YOLOv4 Tiny algorithm. The target frames on the original image are marked with this information and colors are distinguished to indicate different wearing states. The model achieves 94.84%, 96.04% and 94.86% accuracy on three mask wearing states respectively, demonstrating good detection results.

In recent years, ping pong balls have undergone a digital transformation using artificial intelligence techniques. Gomez-Gonzalez S et al. [11] designed a deep conditional generation model. The model maps trajectories to a latent Gaussian distribution space through a network of encoders and decoders, and is trained using stochastic gradient-variant Bayes for fast prediction. The model is able to provide more accurate long-term predictions under low-latency conditions compared to traditional TP methods. Li [12] proposed the combination of Speeded Up Robust Features (SURF) and Long Short-Term Memory (LSTM) algorithms for deep learning to efficiently and accurately analyze and predict ping pong ball rotation trajectories. This method is able to analyze the TP with 96.5% accuracy based on the ball rotation in three coordinate directions. This advancement provides strategic support for table tennis training and competition. Afshar et al. [13] aimed to build table tennis robots that can predict and adapt to the flight path of table tennis balls. The method showed significant improvement in predicting the trajectory and rotational velocity of table tennis balls compared to the traditional modeling methods. Tang et al. [14] utilized a spatio-temporal occlusion paradigm to examine the action perception ability of professional table tennis players. The results showed that professional table tennis players need to perceive the optical flow of the ball flight and focus on information from the opponent’s torso and head to achieve peak performance. Oagaz et al. [15] study explored the use of virtual reality (VR) in table tennis training and its impact on skill learning and transfer. A VR table tennis system was developed combining physical simulation, audiovisual stimulation and motion capture technology to enhance immersion and collect player data. This showed that complex skills can be learned in VR and successfully transferred to the real world.

To summarize, in recent years, the application of YOLOv4 algorithm in object detection has gradually increased, and in waste classification, Saputra APK uses YOLOv4 and its improved version YOLOv4-Tiny model to effectively classify different types of waste. And Xiang X et al. applied YOLOv4 Tiny to engineering vehicle inspection to realize highly efficient intelligent monitoring. In the field of table tennis, the integration of AI technology is increasingly deep, the deep conditional generation model developed by Gomez-Gonzalez team accurately predicts the trajectory of table tennis balls, while Li W combined with deep learning algorithms can efficiently and accurately analyze the rotational trajectory of table tennis balls. In view of this, based on the YOLOv4-Tiny algorithm, the SPM-GRU model was constructed by combining physical motion simulation and machine learning algorithms, which is expected to provide an effective way to predict the trajectory and dynamics of table tennis balls, and bring technical support for training and match analysis.

### Construction of table tennis tp model based on YOLOv4-tiny

To construct an efficient and accurate table tennis ball TP model, the study firstly focuses on the improvement of the YOLOv4-Tiny. In this stage, the Feature Pyramid Network (FPN) module was mainly introduced to enhance the recognition of dynamic features of table tennis balls, thus ensuring that the underlying data for TP is highly accurate and reliable. Subsequently, the study constructed an advanced table tennis ball TP system by fusing the SPM-GRU model.

### Real-time localization of mobile ping-pong balls based on YOLOv4-tiny

In traditional real-time ping-pong localization there are multiple interfering factors such as occlusion, light and target too small, and the problem of high target background complexity and too small area display. YOLOv4-tiny is a lightweight target detection model on the basis of YOLOv4 but more suitable for environments with limited computational resources [16]. It sacrifices a portion of its detection accuracy in exchange for higher operating speed and lower resource consumption, allowing it to perform well in real-time applications and edge computing devices [17]. In view of this, the study introduces the YOLOv4-tiny model to enhance the ability to perform real-time localization of mobile ping pong balls.

**Fig 1:**
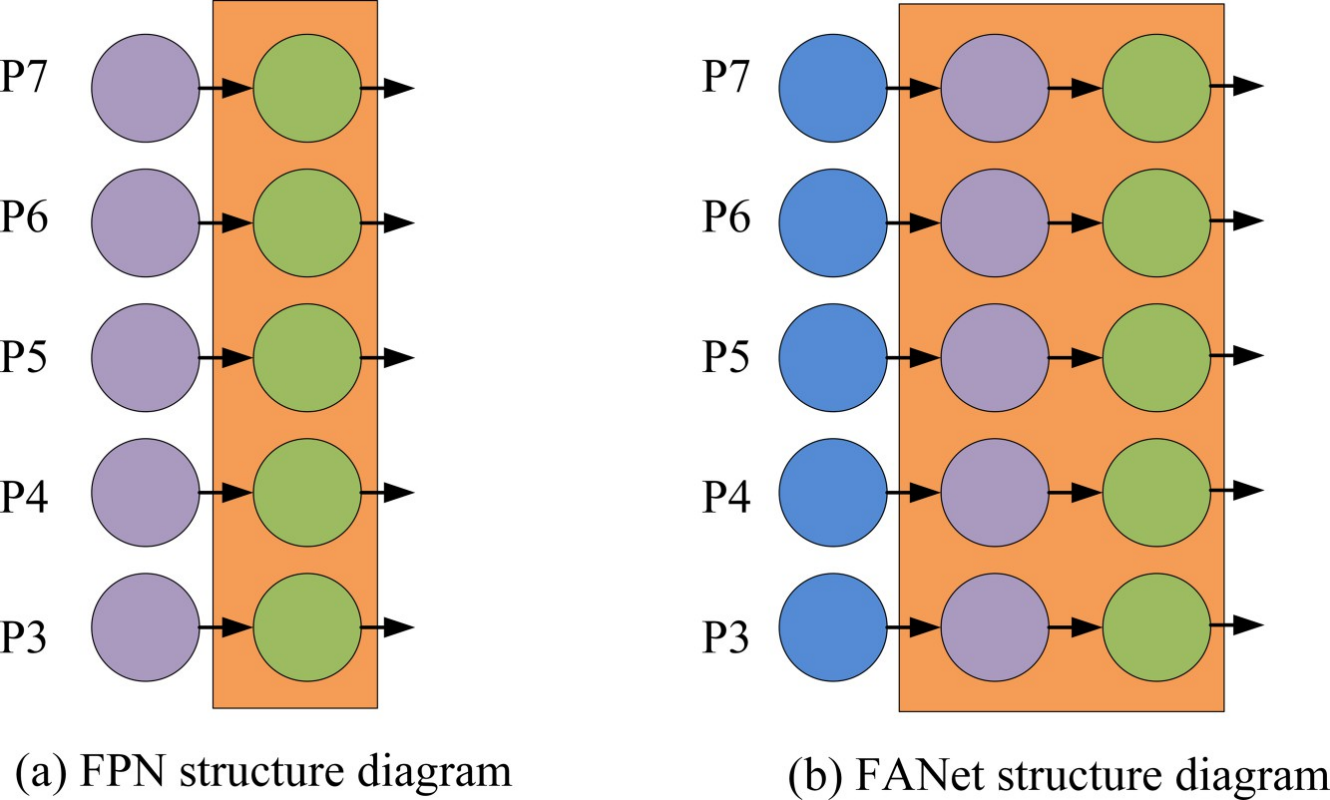
Feature fusion structure diagram.

The selection of YOLOv4-tiny as the primary network structure for the ping-pong ball trajectory prediction model was based on two key considerations. Firstly, although newer versions of YOLO exist, such as YOLOv5 and YOLOv6, which are capable of providing improved accuracy and speed, these versions typically require higher computational resources. YOLOv4-tiny, on the other hand, maintains high recognition accuracy while its lightweight network architecture significantly reduces the demand for computational resources, making it well suited for deployment in real-time applications and edge computing devices, especially in real-time demanding application scenarios such as ping-pong ball trajectory prediction. Secondly, the improvement of the YOLOv4-tiny algorithm, especially the introduction of the FPN and PANet modules, can significantly improve the model’s detail capturing ability and classification accuracy when dealing with table tennis ball recognition. Therefore, although a relatively old version of YOLOv4-tiny was chosen, the targeted optimisation and improvement can ensure that the model maintains high accuracy and stability while meeting real-time processing requirements. To optimize the YOLOv4-Tiny, the FPN module is introduced in it, and its structure is shown in Fig 1.

**Fig 2:**
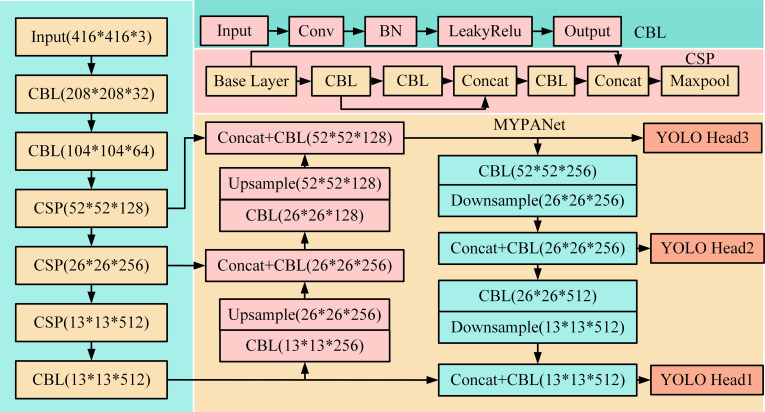
Running process of improved YOLOv4-Tiny.

In Fig 1, the FPN layer fails to effectively utilize the low-level detail information, resulting in poor real-time detection of small or occluded targets. This is mainly due to the fact that the low-level details in the model are not closely combined with the high-level semantic features, and the output details are less. To solve this problem, Path Aggregation Network (PANet) is combined to realize multi-scale fusion and enhancement of features by fusing two paths, bottom-up and top-down. This structure significantly improves the expression ability of YOLO Head in target detection and effectively reduces the influence of complex background. The flow of the improved YOLOv4-tiny is Fig 2.

**Fig 3:**
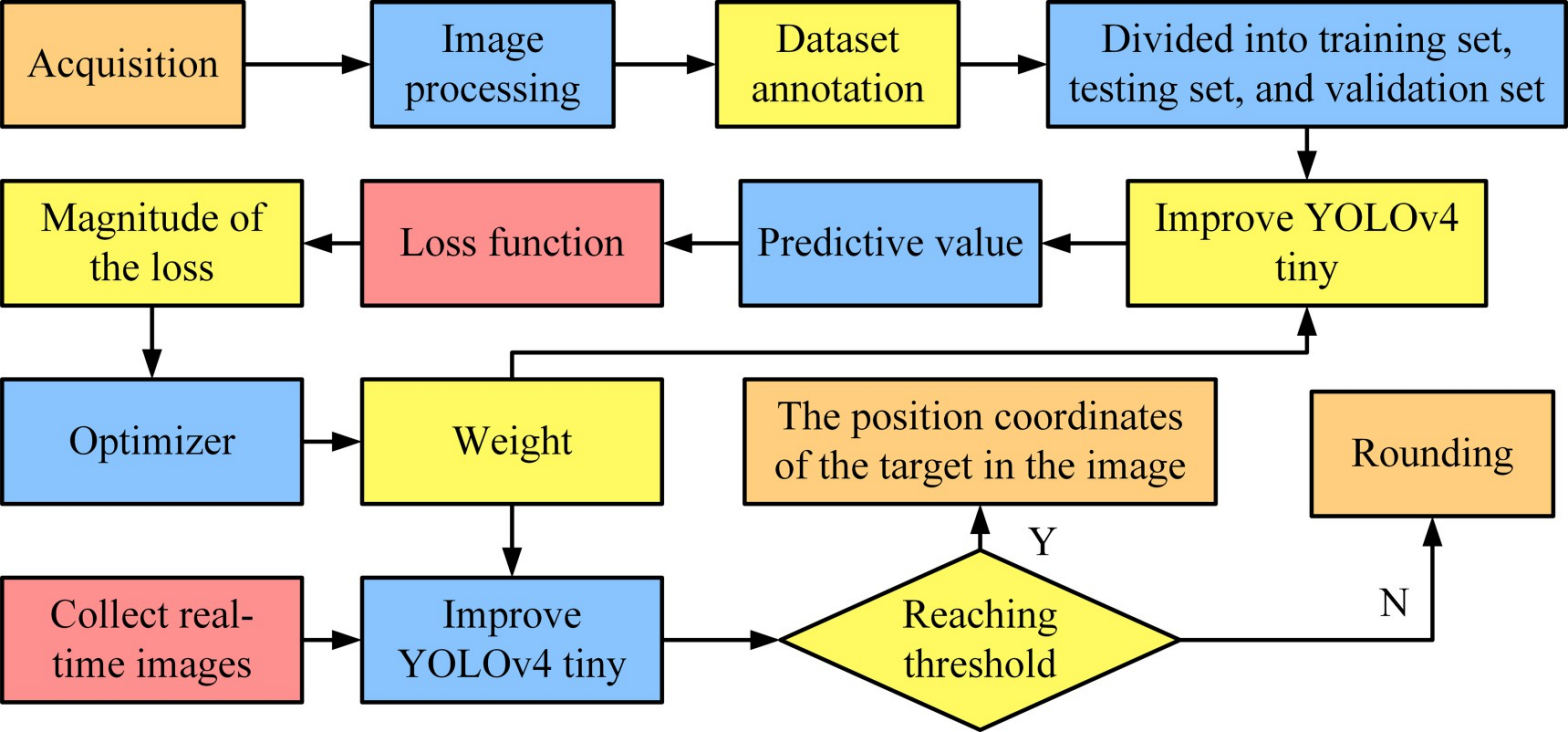
Training steps of the improved YOLOv4-Tiny.

The core of the network architecture illustrated in Fig 2 contains the key components of upsampling, convolution, downsampling, and feature fusion. In Fig 2, the introduction of residual network CSP is aimed at reducing the computational complexity of YOLOv4-Tiny model and improving the training efficiency of the model. In CSP, the first layer uses a convolution kernel with a size of 3x3 and a step size of 1 to extract initial features. Next, the features pass through the two-layer CBL structure, increasing the output graph size from 64 to 128, 256, 512, and the filter size is unified to 3x3 in the process. After each layer, the dimension of the feature map is halved by a maximum pooling layer with a step size of 2 to increase the model’s perception of advanced features. Finally, LeakyReLU is adopted as the activation function in all convolutional layers of the CSP, with a negative slope parameter set to 0.1. LeakyReLU is able to provide a small gradient in the negative region, effectively avoiding the problem of dead neurons that may occur in the training of ReLU activation functions. These components work together to form two main feature fusion processes: upsampling feature fusion and downsampling feature fusion. In the upsampling feature fusion part, layer F1 outputs a feature graph of 13 × 13 × 512, which is changed to 13 × 13 × 256 after processing by the Convolution-BatchNorm-LeakyReLU (CBL) module, and then the size is expanded to 26 × 26 × 256 by upsampling and superposition with the feature graph of layer F2. Finally, the feature map of 26 × 26 × 512 is formed. In the down-sampling feature fusion part, the first down-sampled feature layer is first fused with the F3 layer feature map, which is processed by the CBL module to have a size of 52 × 52 × 256, and further down-sampled to be adjusted to 26 × 26 × 256. This is the same size as the route1, which is superimposed to form the 26 × 26 × 512 feature map, which is then processed by the convolution process to be 26 × 26 × 256. Through this up and down sampling and fusion mechanism, the model is able to better retain and utilize the detailed information of the feature layer [18–21]. The training steps of the improved YOLOv4-Tiny algorithm are shown in Fig 3.

In Fig 3, the development process of the table tennis ball detection model consists of five steps: in the first step, image data containing diverse backgrounds and lighting conditions are collected and accurately labeled to construct a dataset to enhance the generalization capability; in the second step, using the improved YOLOv4-Tiny, the model is trained with these data, and the loss is calculated by comparing the predicted values to the true values and adjusting the weights with the Adam optimizer to improve the accuracy; in the third step, during the training process, the best weights are selected based on the performance of the validation set, and the model is optimized to cope with the new data; in the fourth step, after the training is completed, the test set is used to evaluate the overall efficacy of the model and the ability of ping-pong detection; in the fifth step, in the practical application deployment, the camera is set to capture the real-time image, and the optimized model is used to predict the location of the ping-pong and determine whether the prediction box contains the ping-pong to output the accurate coordinate position, and targets that do not meet the threshold will be discarded.

### Construction of a table tennis TP model based on SPM-GRU

The TP model of the table tennis is affected by many factors, in addition to considering the external factors that are subjected to, it is also necessary to consider the internal factors of the table tennis, such as the change of the physical parameters in the process of operation. The formula for the gravity force on the table tennis is Eq (1) [22, 23].


$$F_{g}=mg$$ (1)

In Eq (1), *m* is usually 0.0027 kg; the value of the acceleration of gravity *g* is specified as 9.8 m/s. The formula for the air resistance of a ping-pong ball is shown in Eq (2).


$$F_{r}=\frac{1}{2}C_{d}\rho Av^{2}$$ (2)

In Eq (2), *C*_*d*_ denotes the drag coefficient; *ρ* denotes the air density; *A* denotes the size of the cross-section; and *v* denotes the size of the velocity direction. The air buoyancy of the table tennis is Eq (3).


$$F_{b}=\frac{1}{6}\rho g\pi D^{3}$$ (3)

In Eq (3), *D* denotes the diameter. The downward motion of the table tennis is analyzed and the expression of the motion on the X-axis is Eq (4).


$$\left\{\begin{array}{l} v_{x}=\frac{v_{x0}m}{kv_{xo}t+m}\\ x=\frac{m}{k}In(1+\frac{kv_{x0}t}{m})+x_{0} \end{array}\right.$$ (4)

In Eq (4), *v*_*x*0_ and *x*_0_ denote the initial velocity and position in the x-axis direction; *x* is the table tennis position in the x-axis. *t* denotes the time of the motion; *v*_*x*_ denotes the velocity in the x-axis direction after *t*; *k* = 0.5*C*_*d*_ρ*A*. The upward motion of the table tennis is analyzed and the expression of the motion on the x-axis is Eq (5).


$$\left\{\begin{array}{l} v_{x}=\frac{-v_{x0}m}{-kv_{xo}t+m}\\ x=-\frac{m}{k}In(1-\frac{kv_{x0}t}{m})+x_{0} \end{array}\right.$$ (5)

In Eq (5),—indicates that the direction is opposite to Eq (4). The expression of the motion in the horizontal direction X and Y axes is consistent. The upward motion of the table tennis is analyzed, and the expression of the motion in Z-axis is Eq (6).


$$\left\{\begin{array}{l} v_{z}=\sqrt{\frac{mg}{k}}\tan\left[-\sqrt{\frac{kg}{m}}t+\arctan(v_{z0}\sqrt{\frac{k}{mg}})\right]\\ z=\frac{m}{k}In\left(\left|\cos\left(\sqrt{\frac{kg}{m}}t\right)+v_{z0}\sqrt{\frac{kg}{m}}\sin\left(\sqrt{\frac{kg}{m}}t\right)\right|\right)+z_{0} \end{array}\right.$$ (6)

In Eq (6), *v*_*z*0_ denotes the initial velocity in the Z-axis; *z* denotes the position of the ping-pong in the Z-axis; *v*_*z*_ denotes the velocity in the Z-axis after *t*; *z*_0_ denotes the initial position point of the ping-pong in the x-axis. The downward motion of the table tennis is analyzed and the expression of the motion on the Z-axis is Eq (7).


$$\left\{\begin{array}{l} v_{z}=\sqrt{\frac{mg}{k}}\frac{1-e^{2t\sqrt{\frac{mg}{k}}}}{1+e^{2t\sqrt{\frac{mg}{k}}}}+v_{z0}\\ z=-\frac{m}{2k}In\frac{{\left(1+e^{2t\sqrt{\frac{mg}{k}}}\right)}^{2}}{4e^{2t\sqrt{\frac{mg}{k}}}}+z_{0}+v_{z0} \end{array}\right.$$ (7)

The modeling based on the above equation is Eq (8).


$$\left\{\begin{array}{l} v_{xout}=k_{x}•v_{xin}+b_{x}\\ v_{yout}=k_{y}•v_{yin}+b_{y}\\ v_{zout}=k_{z}•v_{zin} \end{array}\right.$$ (8)

In Eq (8), *k*_*x*_, *k*_*y*_ and *k*_*z*_ are the parameters before the rebound; *b*_*x*_ and *b*_*y*_ are the parameters after the rebound; *v*_*xin*_, *v*_*yin*_ and *v*_*zin*_ are the input velocities; *v*_*xout*_, *v*_*yout*_ and *v*_*zout*_ are the output velocities. Conventional physical models usually rely on measuring a series of parameters, thus ignoring the characteristic information embedded in the long-term time series. The GRU model is a RNN variant designed to address the problem of vanishing or exploding gradients encountered by conventional RNNs when dealing with long sequential data. In view of the excellent performance demonstrated by the GRU model in dealing with long-term time series prediction, the study proposes a novel TP model: the Trajectory Prediction Model Combining Physical Motion Model and Enhanced Contextual Information (SPM-GRU). In the GRU model, the formula for its update gate is Eq (9).


$$z\left(t\right)=sigmoid\left(W\_z*\left[h\left(t-1\right),\;x\left(t\right)\right]+b\_z\right)$$ (9)

In Eq (9), *z*(*t*) indicates the part of the control hidden state that needs to be updated. Its value ranges from 0 to 1; *W*_*z* denotes the weight matrix; *h*(*t*-1) denotes the hidden state at the previous moment; *x*(*t*) denotes the current input state; *b*_*z* is the bias term. The formula for reset gate is Eq (10).


$$r\left(t\right)=sigmoid\left(W\_r*\left[h\left(t-1\right),x\left(t\right)\right]+b\_r\right)$$ (10)

In Eq (10), *r*(*t*) controls how to combine the hidden state of the previous moment with the input of the current moment to generate the candidate hidden state; *W*_*r* denotes the weight matrix of the reset gate; *b*_*r* is the bias term in the reset gate. The formula for the candidate hidden state is Eq (11).


$$h∼\left(t\right)=tanh\left(W\_h*\left[r\left(t\right)*h\left(t-1\right),x\left(t\right)\right]\right)$$ (11)

In Eq (11), *h*∼(*t*) is the candidate hidden state based on the current input and the hidden state in the previous moment; *W*_*h* is the weight matrix in this state. The expression of the current moment hidden state is Eq (12).


$$h\left(t\right)=\left(1-z\left(t\right)\right)*h\left(t-1\right)+z\left(t\right)*h∼\left(t\right)$$ (12)

**Fig 4:**
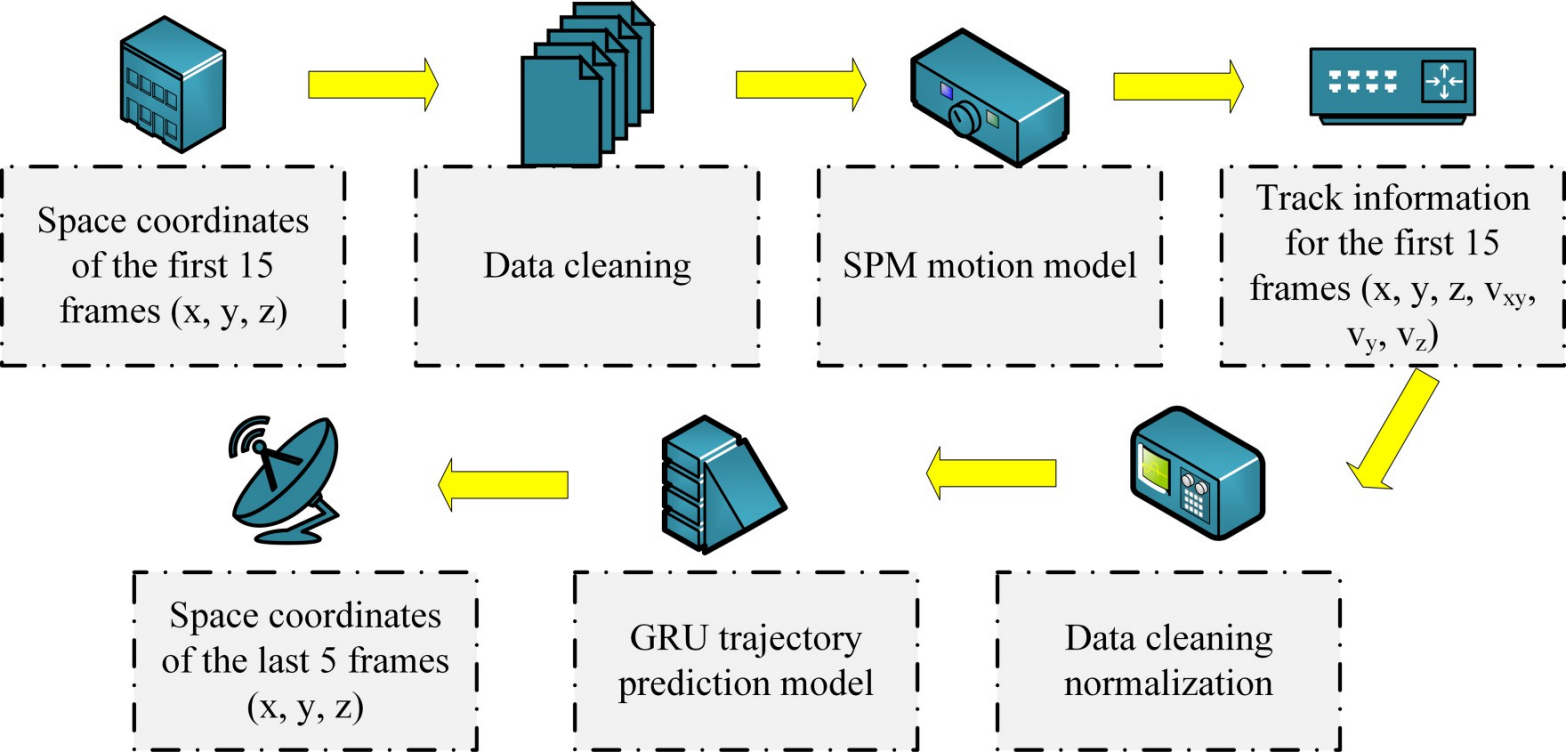
Flowchart of SPM-GRU model operation.

In Eq (12), *h*(*t*) denotes the final hidden state at the current moment; (1-*z*(*t*)) and *z*(*t*) denote the parts to be discarded and retained, respectively [24, 25]. In this way, the GRU network is able to decide which information should be kept or forgotten in a new step. The study constructs the SPM-GRU model as shown in Fig 4.

**Fig 5:**
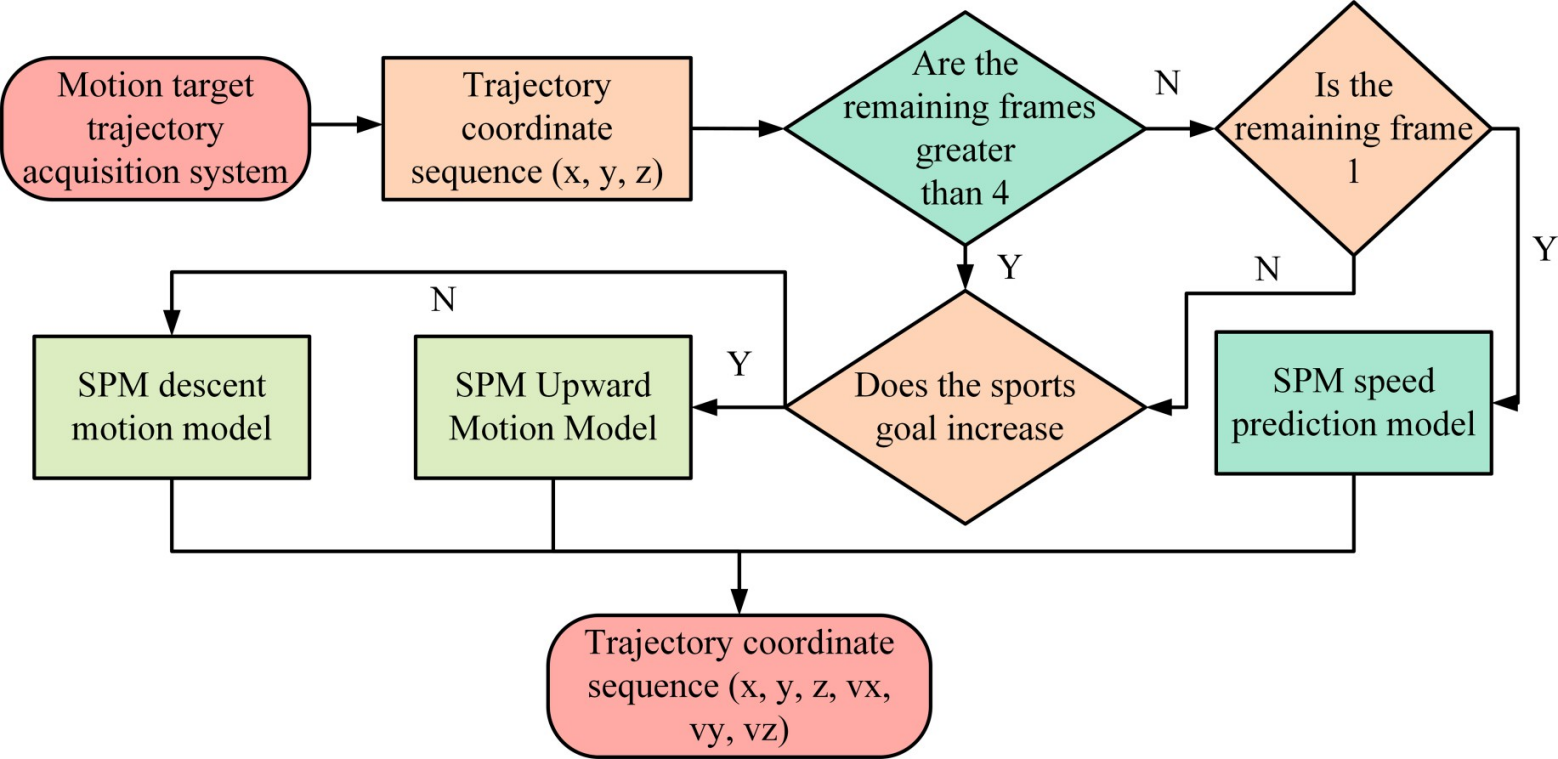
Data expansion diagram of PM-GRU model.

In the SPM-GRU model shown in Fig 4, the starting step of the model processing is to receive the first 15 frames of 3D spatial coordinate data captured by the ping-pong ball trajectory acquisition system. These data are first subjected to data cleaning, and the cleaned 3D time series data (x, y, z) are then fed into a specially designed SPM motion model. The SPM model serves to analyze and process each frame of data in depth, not only retaining the original position information, but also adding three extra dimensions: velocity vectors, thus expanding the data from three to six dimensions. Then, the expanded six-dimensional time series data (x, y, z, v_x_, v_y_, v_z_) are normalized to ensure that the data have a uniform scale and proportion in different dimensions, which enables the GRU model to process these data more efficiently. The task of the GRU model is to predict the spatial location of the ping-pong in the next five frames based on these processed input data. PM-GRU Model Data Expansion The steps are shown in Fig 5.

**Fig 6:**
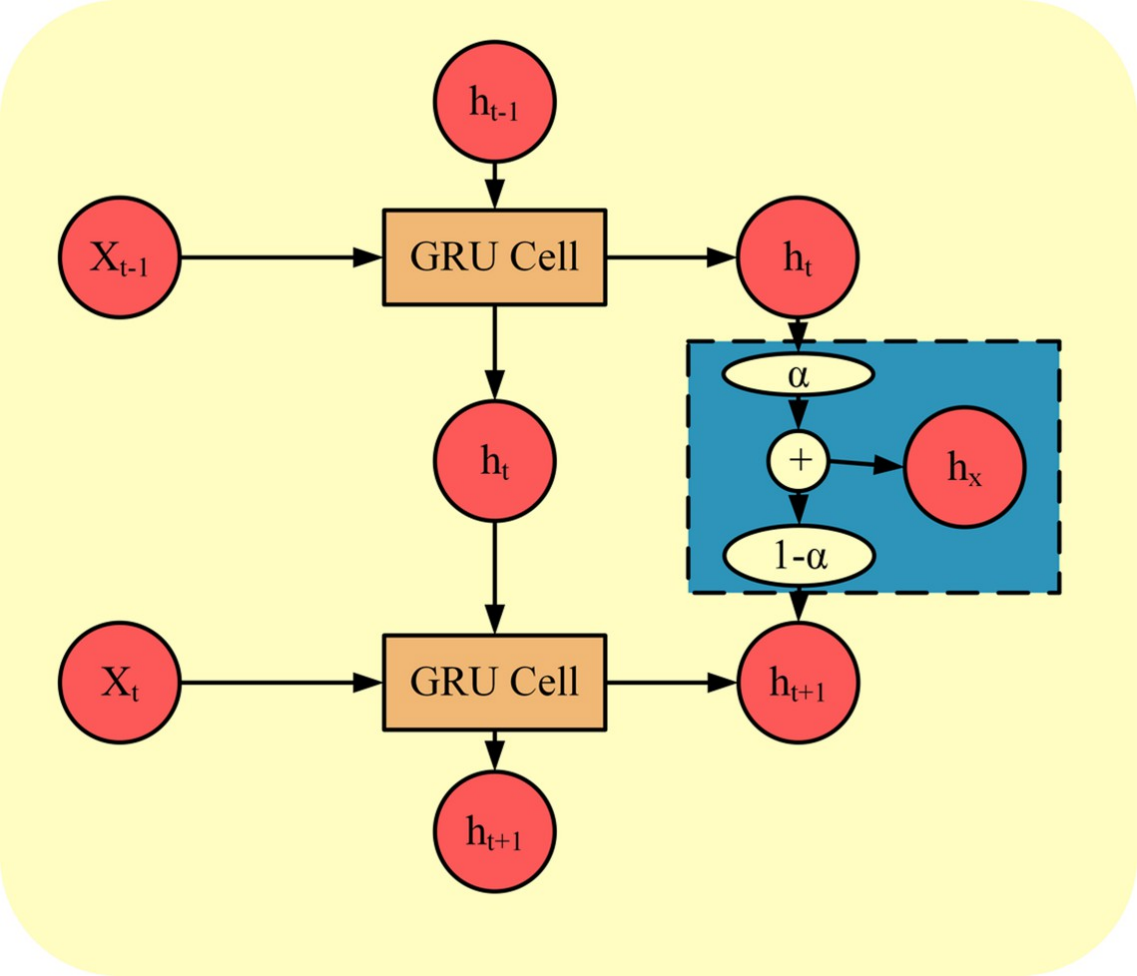
Improved GRU prediction unit structure diagram.

Fig 5 depicts a complex processing flow for a table tennis ball trajectory that converts the original 3D trajectory coordinate sequence into a 6D sequence to provide a more detailed analysis. The process first checks the number of frames contained in the coordinate sequence obtained through the trajectory acquisition system. If the number of frames exceeds four, the system analyzes whether the coordinates are ascending or descending and selects the appropriate motion model to process the data accordingly. When processing to the last frame of coordinates, the system uses a velocity prediction model. In addition, the processing flow incorporates a GRU network model to improve the long and short time series memory problem, which results in higher prediction accuracy. The improved GRU prediction unit structure is shown in Fig 6.

Fig 6 demonstrates the internal loop structure of the GRU. In the standard GRU model, the hidden state of the next moment *h*_*t*_ is mainly controlled by the reset gate and update gate, but the connection between the hidden states of neighboring moments is not fully considered. To optimize this problem, the study proposes an improved GRU structure by fusing the short-time information of adjacent frames. The specific method is to introduce the hyper-parameter *α*, as a linearly weighted coefficient, to weight and fuse the hidden states of the current moment and the previous moment, and this method enhances the model’s ability to capture time series data.

## Performance evaluation and discussion of table tennis TP models

To verify the applicability and superiority of the research constructed prediction model, the performance of the improved YOLOv4-Tiny algorithm chosen for the model is first tested. Then, the performance of the human body estimation recognition algorithm is tested. Finally, the functional modules of the fixed-point shooting hit prediction system are tested.

### Performance test of improved YOLOv4-Tiny algorithm

**Fig 7:**
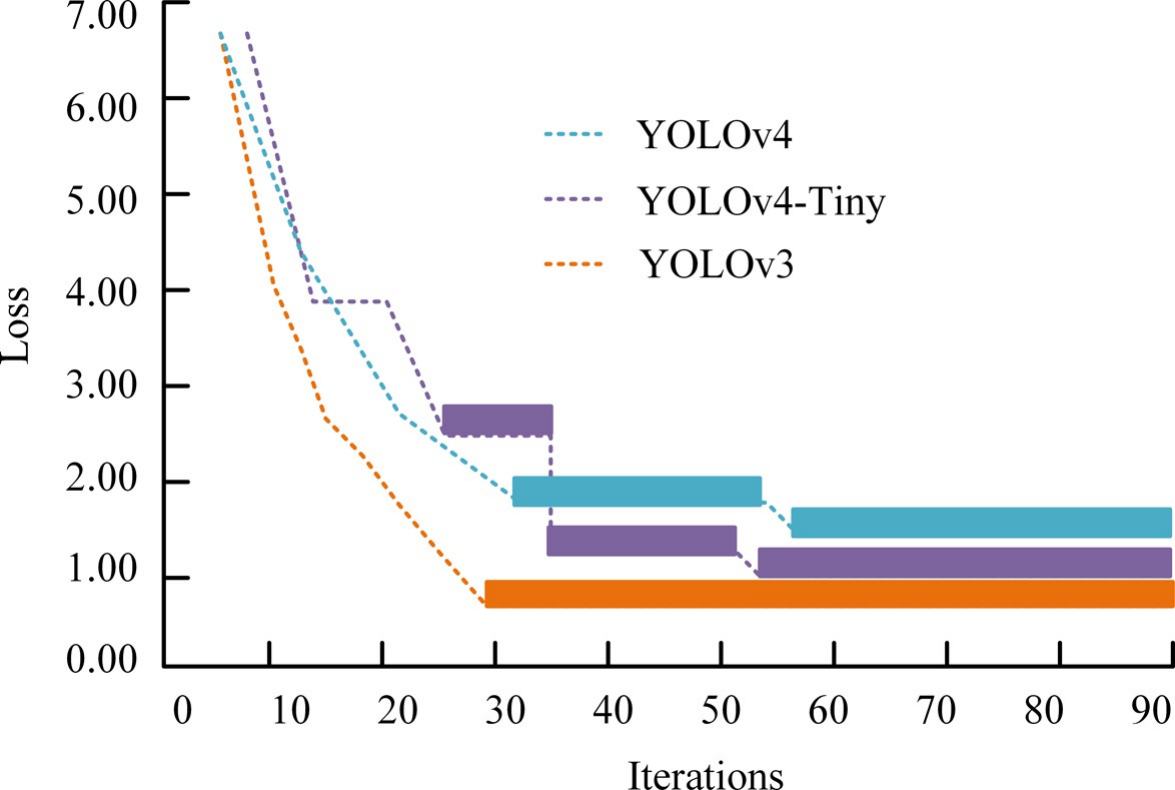
Loss variation curves of three models.

The study conducted experiments with uniform hardware and software configurations to eliminate the effect of hardware differences on the results, and the experiments were conducted using CentOS 8 operating system and Python 3.9 to maintain the consistency of the environment and programming efficiency. The deep learning framework chosen was TensorFlow, combined with CUDA 11.4 to utilize the computational power of NVIDIA RTX3080 GPUs and Intel Core i9-10900K CPUs. It is paired with 64 GB of RAM and 32 GB of graphics memory to support complex model training. Visual Object Classes (VOC) 2007 is a widely used computer vision dataset provided by the Visual Object Classes program. To verify the superiority of the YOLOv4-Tiny, YOLOv3 and YOLOv4 are compared, and the loss curves of the three models are displayed in Fig 7.

**Fig 8:**
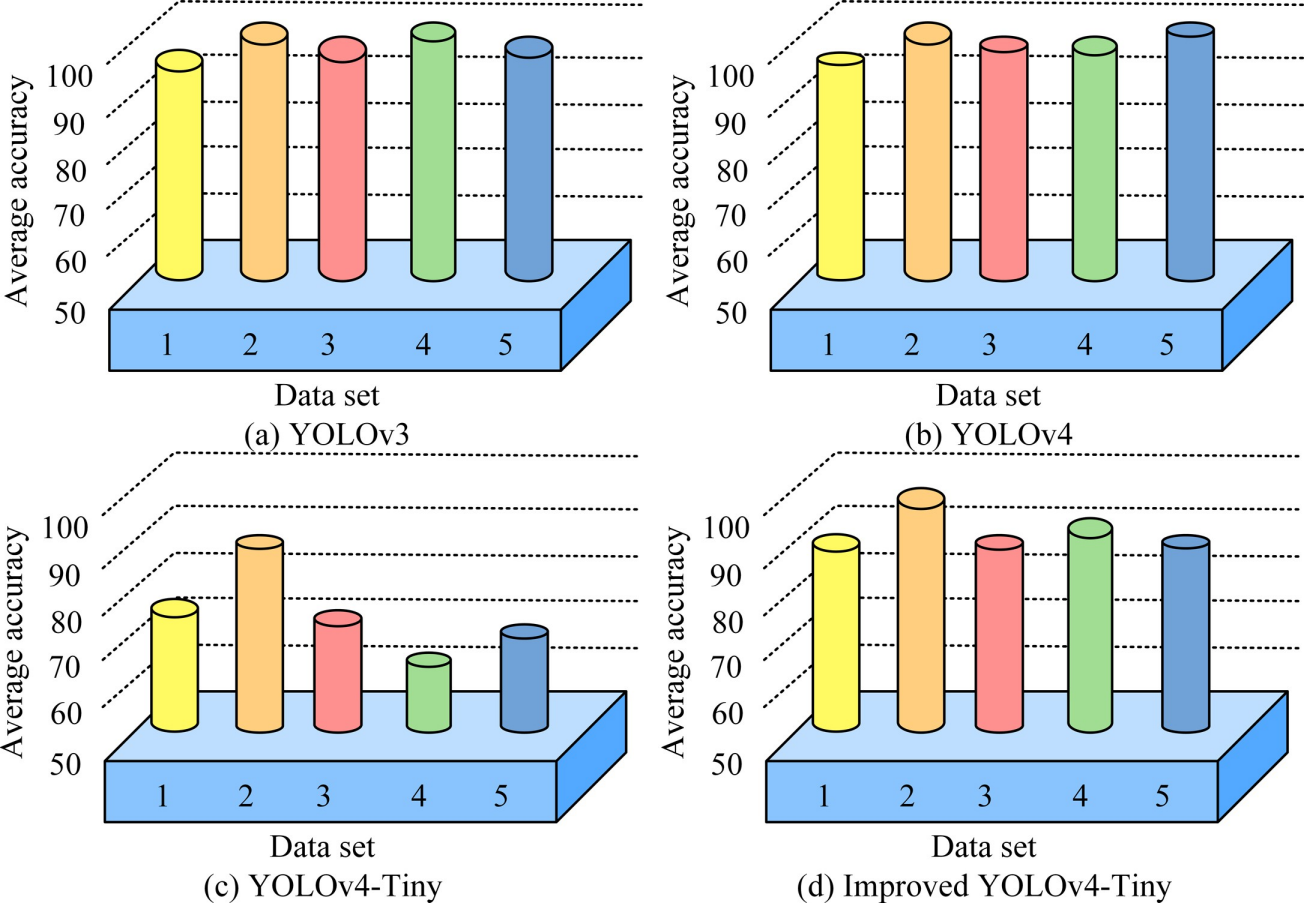
Average accuracy of different models.

Fig 7 shows the curves of loss function value changes on the training set for the three different models. In the initial 30 iterations, the loss decreases slowly due to the freezing of some parameters of the model backbone network. After more than 30 iterations, the backbone network unfreezes and participates in the training, leading to a significant decrease in the loss. YOLOv3 exhibits the lowest loss values among these models, but YOLOv3 has the disadvantage of potentially overlooking errors caused by errors in the prediction frame aspect ratio. In addition, both YOLOv4 and YOLOv4-Tiny show stable convergence performance, with YOLOv4-Tiny performing the best, with its loss value reduced to 1.54, revealing the superiority of its training process and optimization results. To verify improved YOLOv4-Tiny, the comparison with the three models mentioned above is continued, and the comparison of the average accuracy of the four models is shown in Fig 8.

Fig 8 illustrates the dataset divided into five categories: vehicles, animals, humans, furniture, and others, denoted by the numbers 1 to 5, respectively. The YOLOv4 and YOLOv3 models demonstrate excellent performance on these categories, with average accuracies of more than 90% for each, proving their high performance in target recognition. In contrast, YOLOv4-Tiny, a lightweight model, shows relatively weak performance on these categories with an average accuracy of only 64.38%. However, the improved YOLOv4-Tinysignificantly improves the recognition accuracy, achieving an average accuracy of 86.74%, which is a 22.36% improvement over the original YOLOv4-Tiny. This significant performance improvement is due to the inclusion of PANet, which enhances the model’s ability in detailed classification and accurate prediction, demonstrating that the introduction of an advanced network structure into a lightweight model can greatly perfect the performance of the target detection task.

**Fig 9:**
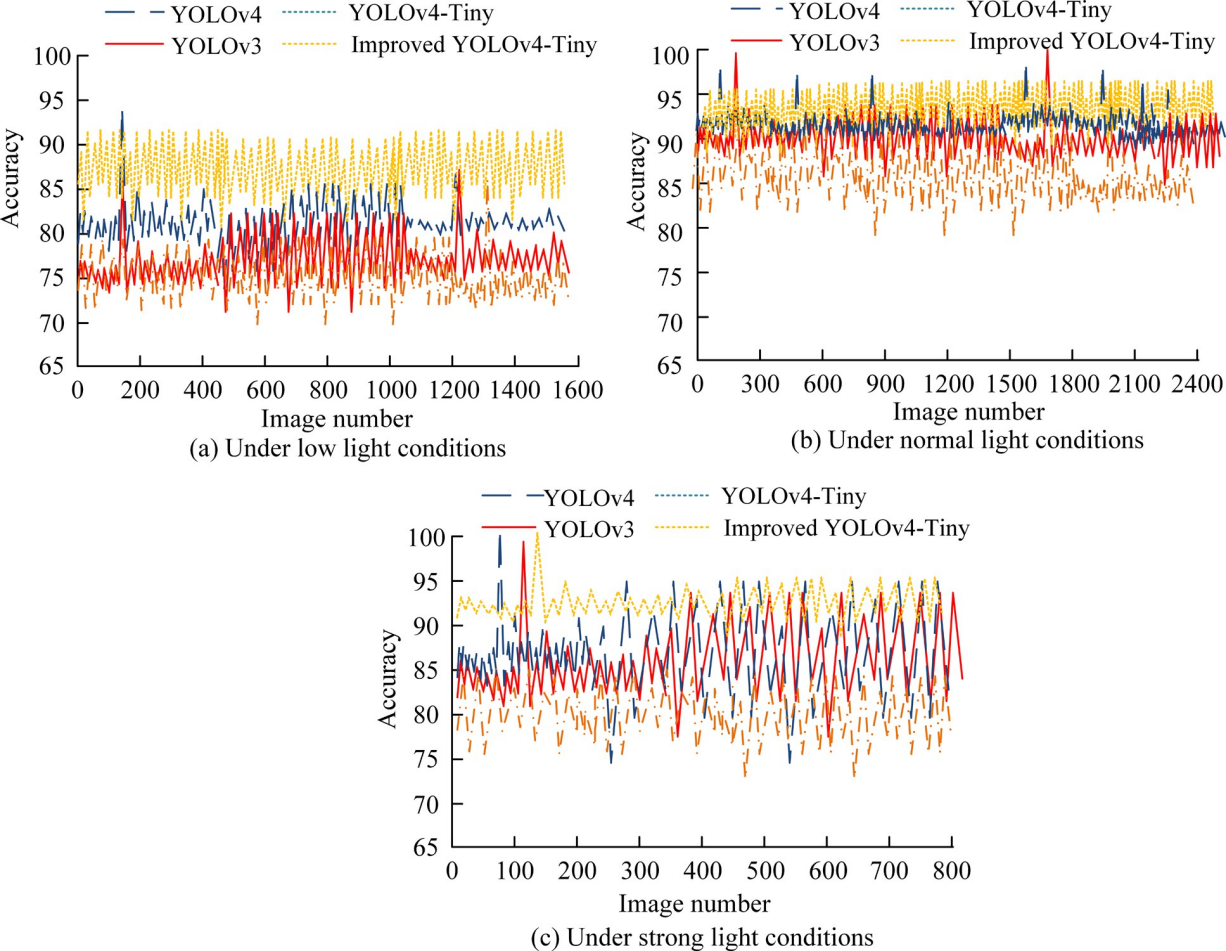
Comparison of detection accuracy of table tennis balls using different models.

The data set used in the study mainly consists of high-definition video data collected from a number of different table tennis match and training scenarios. The dataset consists of about 10,000 video clips, each about 10 seconds in length and at a resolution of 1920x1080, with a total of about 200,000 frames labeled, in which the position and status of the ping-pong ball in each frame are precisely labeled. The entire video dataset covers the speed and rotation of table tennis in a variety of lighting conditions and different backgrounds, and the videos in the dataset come from players at different levels, including amateur, semi-professional and professional levels, ensuring a variety of technical movements. The original video data is pre-processed, including noise removal, color correction and size adjustment, to ensure the quality and consistency of the input data. The pre-processed data set is divided into training sets, test sets, and validation sets in an 8:1:1 ratio, ensuring that the model can be trained and validated on a wide range of data, while also fairly evaluating model performance on independent test sets. First, the data set was classified according to weak light, normal light and strong light, and the ping-pong ball detection accuracy results of different algorithms were obtained, as shown in Fig 9.

The results in Fig 9 reveal the performance differences of the four models under various lighting conditions. In the low-light environment (Fig 9A), the average accuracy of all models does not exceed 90%, but the research model reaches 89.12%, which is a 24.97% improvement over the original YOLOv4-Tiny, showing excellent low-light processing capability. Under normal light conditions (Fig 9B), the accuracy of the research model, YOLOv3 and YOLOv4 all improved, with the research model leading the way with 94.35%, demonstrating its effectiveness in standard light environments. In the strong light environment (Fig 9C), the research model demonstrated smaller fluctuations in accuracy, with an accuracy of 93.46%, which is superior to the other models, proving that it maintains high stability and superior performance under extreme light conditions. Continuing to compare the performance of the above models, the metrics frame rate (FPS), Recall, Precision, Accuracy, and F1-score are introduced. Table 1 shows the results.

**Table 1:** Comparison of different models on different evaluation indicators.

Method	Precision (%)	Accuracy (%)	F1-score (%)	FPS (Frame/s)	Recall (%)
YOLOv4-Tiny	86.31	88.67	87.54	123	89.46
YOLOv3	85.34	87.15	87.33	48	85.41
YOLOv4	91.51	93.34	95.36	35	94.28
Research model	95.17	94.86	96.54	85	98.41

The data comparisons in Table 1 show the performance differences between YOLOv3, YOLOv4, YOLOv4-Tiny, and the improved YOLOv4-Tiny models in terms of precision, recall, and detection speed. YOLOv4 achieves 6.17% and 8.87% improvement in precision and recall, respectively, compared to YOLOv3, which indicates that YOLOv4 has been significantly optimized in terms of significant optimization in feature extraction and target detection accuracy. However, this optimization comes at the expense of the detection speed with a frame rate of only 35 frames/sec, reflecting the trade-off between complexity and speed. Further, the YOLOv4-Tiny version leads to a decrease in precision and recall while simplifying the network structure to improve detection speed. This phenomenon may stem from the fact that its simplified backbone network attenuates the feature extraction capability and performs especially poorly in the detection of small target objects. Finally, the improved YOLOv4-Tiny improves the precision and recall by 3.66% and 4.13%, respectively, relative to the original YOLOv4, while the detection speed is significantly enhanced to 85 frames/sec. The improved YOLOv4-Tiny maintains accurate recognition of details while efficiently processing real-time image data, showing its effective balance between fast and accurate detection.

### Performance test of SPM-GRU ping-pong ball TP model

**Fig 10:**
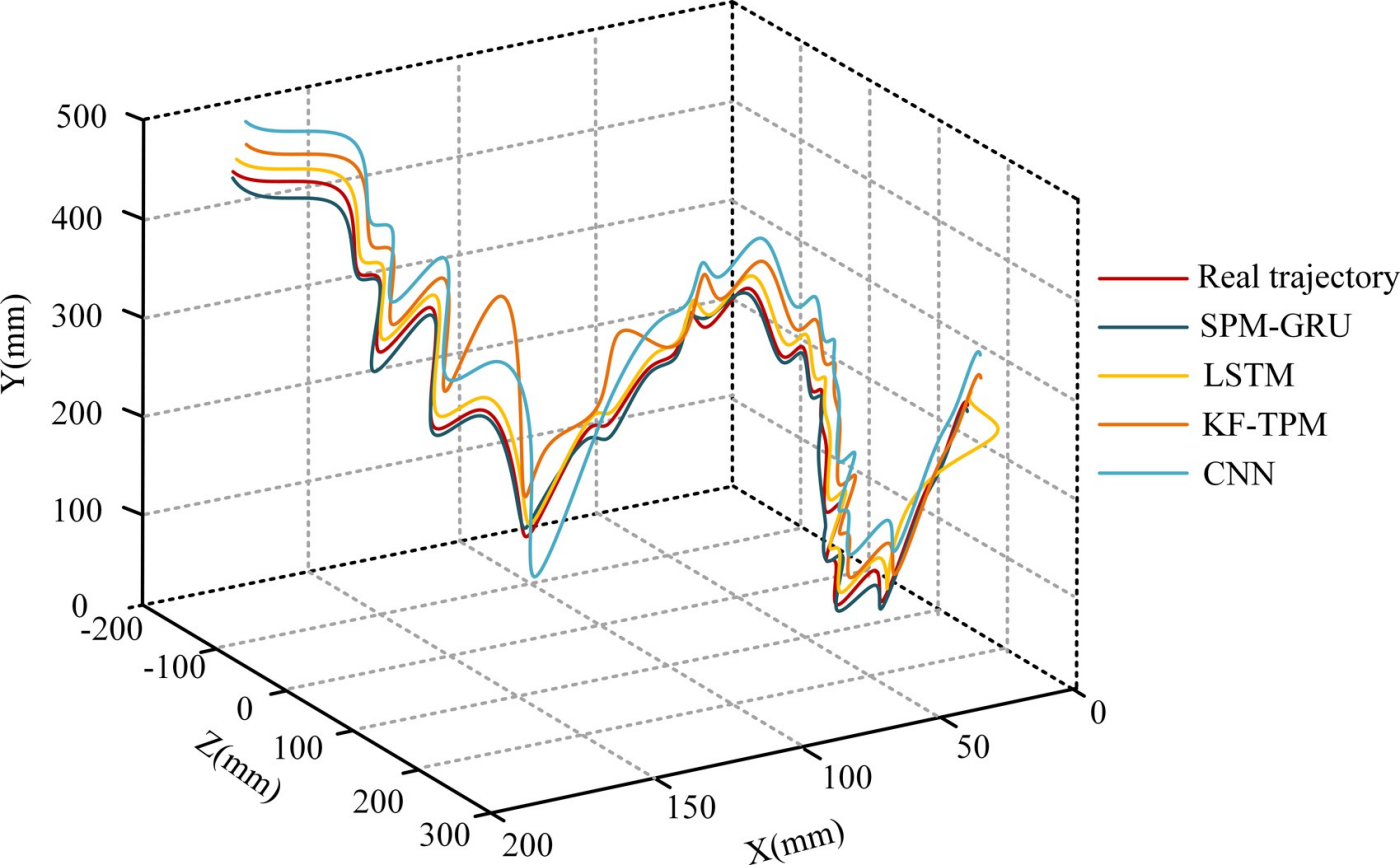
Comparison between three algorithms for predicting trajectories and real trajectories.

To verify the superiority of constructing SPM-GRU ping-pong TP model in the study, LSTM model, Convolutional Neural Network (CNN) model, Kalman Filter-based Trajectory Prediction Model (KF-TPM) for comparative analysis. The four models are applied to the real scenario of table tennis TP, and the comparison between the predicted trajectories and the real trajectories of the four models is shown in Fig 10.

**Fig 11:**
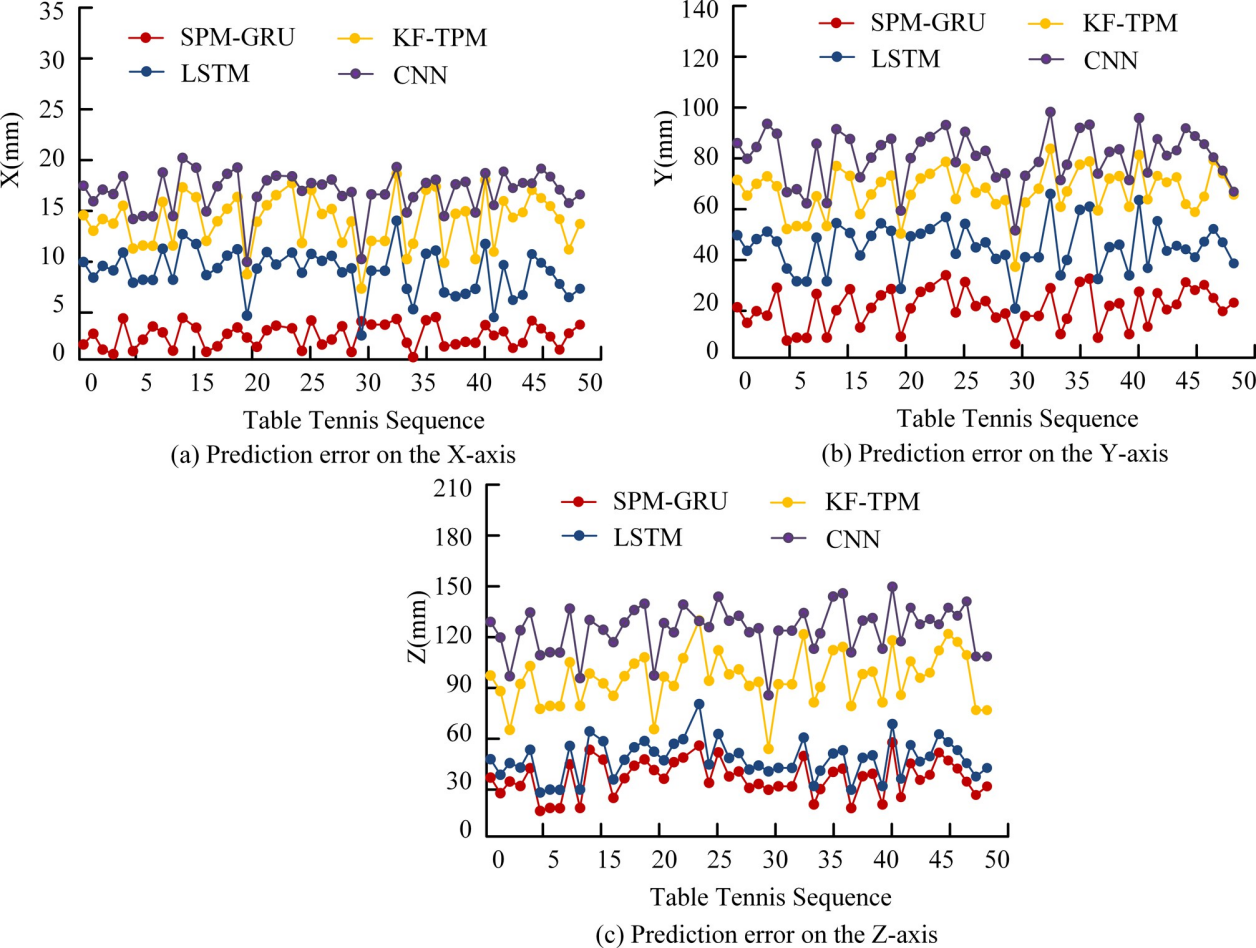
Comparison of errors on the coordinate axis.

In Fig 10, the table tennis ball TP results of each model are represented by different color lines, in which the SPM-GRU model is represented by a green line, and the LSTM model, CNN model, and KF-TPM are represented by yellow, orange, and blue lines. The CNN and the KF-TPM have a large discrepancy between the prediction of table tennis ball trajectory and the real trajectory, especially in the trajectory’s center part. This may reflect the limitations of these two models in dealing with fast movements and complex dynamics of table tennis balls. The SPM-GRU model and the LSTM model showed high accuracy in predicting the trajectories of table tennis balls. In contrast, the LSTM model, although it performed well in the overall TP, suffered from some prediction bias at the end of the movement. The results clearly demonstrate the superiority of the SPM-GRU model in table tennis ball TP compared to other models, especially in the accuracy of predicting trajectories and the ability to fit the real trajectories. The trajectories of 50 table tennis balls were predicted and the errors of each model in X, Y and Z axes are exhibited in Fig 11.

Fig 11(A) presents the prediction errors of the models on the X-axis. In this dimension, the errors of all models are relatively small, fluctuating between 0 and 20 mm. The SPM-GRU model performs well on this metric, with its error controlled within 5 mm. Fig 11(B) demonstrates the prediction error on the Y-axis, where the error of each model fluctuates between 0 and 100 mm, with the SPM-GRU model’s error being effectively controlled within 30 mm. The data in Fig 11(C), on the other hand, focuses on the error on the Z-axis. In this more complex dimension, the errors of the models fluctuate widely, between 0 and 150 mm. the SPM-GRU model and the LSTM model show relatively small errors, fluctuating around 30 mm. Especially, the SPM-GRU model shows an average error of 4.6 mm, 25.3 mm and 35.58 mm in X, Y and Z axes, respectively, which fully verifies its superiority in comprehensive prediction accuracy.

In order to further prove that the table tennis trajectory prediction model constructed in this study has better practical application, the study introduces reference [26] and reference [27] for comparison, in which a tennis trajectory prediction model and a basketball trajectory prediction model are proposed in [26, 27], respectively. Three different table tennis running trajectories, i.e., serve arc trajectory, bounce arc trajectory and middle straight line trajectory, are selected to test the prediction accuracy and prediction time of each type of model, as shown in Table 2.

**Table 2:** Prediction accuracy and prediction time of different models.

Shooting environment	Test index	CNN	KF-TPM	LSTM	Reference [26]	Reference [27]	SPM-GRU
Service arc trajectory	Prediction time /s	1.6s	1.4s	1.1s	0.5s	0.6s	0.2s
	Prediction accuracy /%	85.5%	87.3%	89.6%	95.2%	94.5%	99.5%
Bounce arc trajectory	Prediction time /s	2.3s	1.8s	1.4s	0.8s	0.9s	0.5s
	Prediction accuracy /%	86.4%	88.7%	90.2%	96.3%	95.4%	98.6%
Straight line trajectory in the middle	Prediction time /s	1.5s	1.1s	0.9s	0.6s	0.3s	0.1s
	Prediction accuracy /%	88.1%	89.7%	90.5%	96.5%	97.3%	99.2%

Table 2 shows the predictive performance of six models under three different ping pong ball running paths. As can be seen from Table 2, compared with the three basic models CNN, KF-TPM and LSTM, the SPM-GRU model proposed in reference [26], reference [27] and the text has better prediction effect. The prediction accuracy of SPM-GRU is the highest among the three types of serving arc trajectory, bouncing arc trajectory and middle straight trajectory, which are 99.5%, 98.6% and 99.2% respectively. In addition, the prediction time of SPM-GRU is as low as 0.1s. The two models in reference [26, 27] also have good performance in table tennis trajectory prediction tasks, which indicates that the trajectory prediction method designed by the research may be beneficial to other sports such as table tennis.

## Conclusion

To improve the accuracy of table tennis TP, the study is based on YOLOv4-Tiny to construct SPM-GRU table tennis TP model. In the comparison with YOLOv3, YOLOv4, and YOLOv4-Tiny algorithms: YOLOv4-Tiny performs the best, and its loss value is reduced to 1.54; in the recognition experiments of multi-targets, the improved YOLOv4-Tiny significantly improves the recognition accuracy and achieves 86.74% average accuracy, which is 22.36% higher than that of the original YOLOv4-Tiny by 22.36%; In ping-pong recognition experiments, the improved YOLOv4-Tiny has the best performance under different lighting conditions, especially in low light, reaching 89.12% accuracy, which is significantly better than other models, and maintains the high stability and accuracy under normal and strong light; the improved YOLOv4-Tiny has good performance in all aspects, and the processing efficiency reaches 85 frames/s. In comparison with the LSTM, the modified YOLOv4-Tiny model significantly improves recognition accuracy, reaching 86.74% average accuracy, which is better than the original YOLOv4-Tiny model by 22.36%. In comparison with the LSTM model, CNN model and KF-TPM, the SPM-GRU model predicts the trajectory with the highest degree of fit to the real trajectory of the table tennis and has the smallest error in X, Y and Z axes, with an average error of 4.5 mm, 25.3 mm and 35.58 mm, respectively. The results illustrate that the research model can not only accurately recognize ping-pong in multiple environments, but also adapt to different practical application scenarios, showing good generalization ability and practical value. However, there are still shortcomings in this research, due to the many parameters of the neural network model and the complexity of computation, which can easily lead to the lack of significant memory, especially in the case of multi-model deployment, model compression techniques need to be used to reduce the cost and computational requirements. Further improvements will be made in future research.

## Future work

Although this study has achieved remarkable results in the prediction of table tennis trajectory, there are still some limitations in this study, and the following aspects can be improved in future work. First, there is still room for improvement in the current model’s performance when dealing with extreme lighting and fast-moving targets. Future research may plan to introduce some graphics processing techniques with better performance under extreme conditions to further optimize the robustness of the algorithm. Second, the current research mainly focuses on the trajectory prediction of table tennis, and future studies can collect data of other sports types and combine the characteristics of different sports to verify the generalization of the model in other sports. Third, future research can also explore the integration of multi-modal information such as sound, image and motion data, so as to improve the recognition accuracy and prediction ability of table tennis motion state.
